# Supplementary material for: Transition path times of coupled folding and binding reveal the formation of an encounter complex
Source: Nat Commun. 2018 Nov 9;9:4708. doi: 10.1038/s41467-018-07043-x (PMC6226497; doi:10.1038/s41467-018-07043-x)
Supplement: Supplementary file 1 — Supplementary Information [file 41467_2018_7043_MOESM1_ESM.pdf]

Supplementary Information for

"Transition path times of coupled folding and binding reveal the formation of an encounter complex"

Sturzenegger et al.

Supplementary Figures

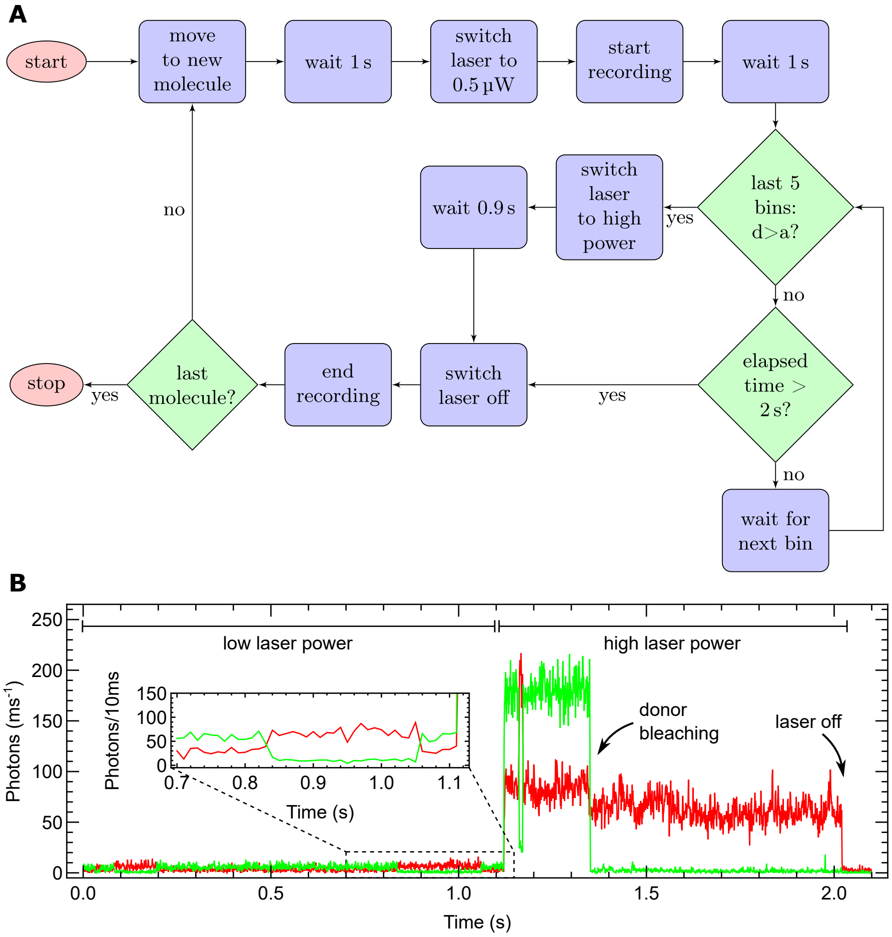

Supplementary Figure 1: Data acquisition procedure for immobilized molecules. (**A**) Flowchart depicting the logic used to automatically acquire photon time traces. (**B**) Example time trace of an immobilized molecule, showing the switch between low and high laser power. Acceptor fluorescence is shown in red and donor fluorescence in green.

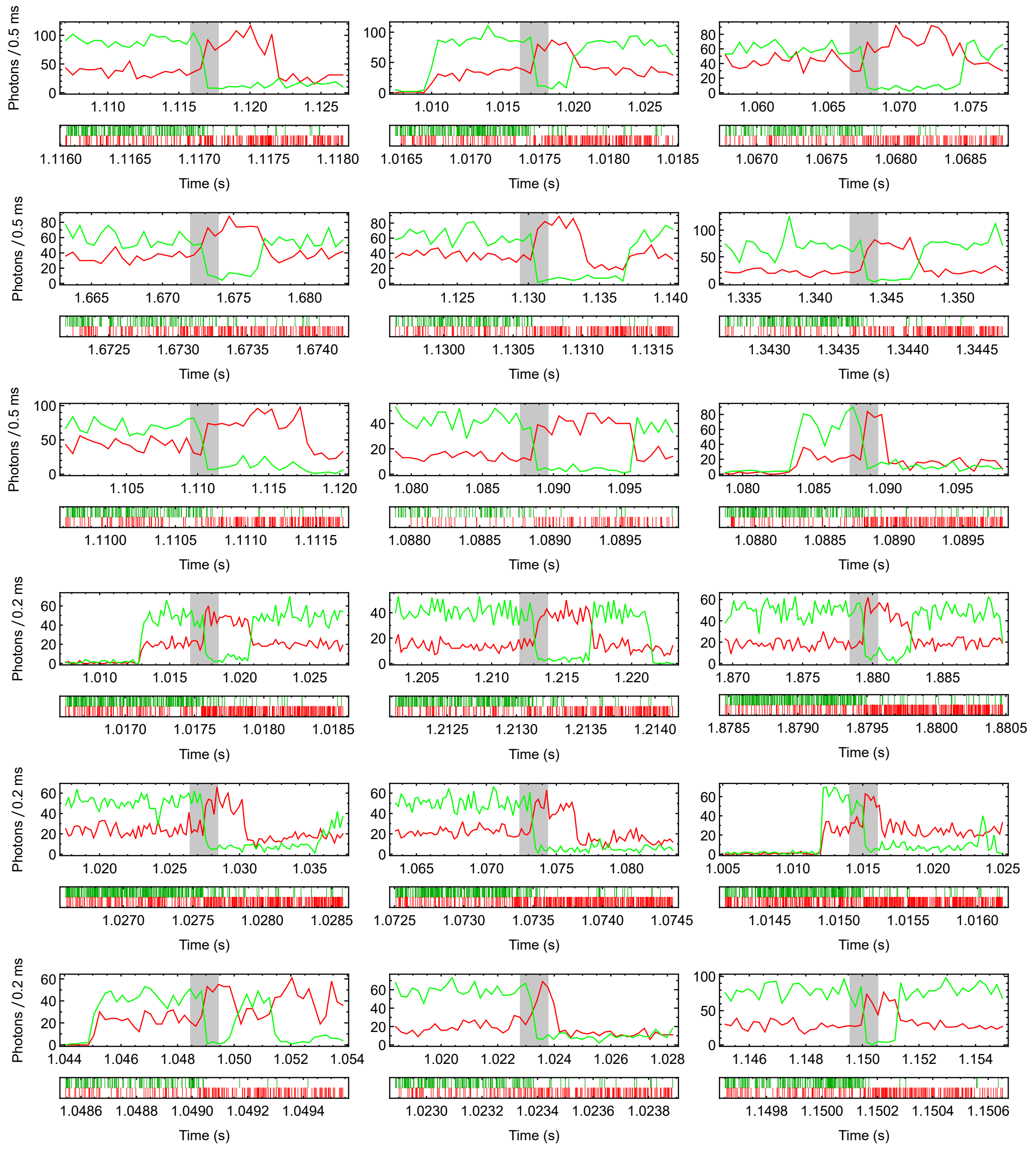

Supplementary Figure 2: Example time traces, with donor fluorescence in green and acceptor fluorescence in red. Below each binned trace, the individual photon arrival times are plotted for the grey shaded regions, which contain the binding events.

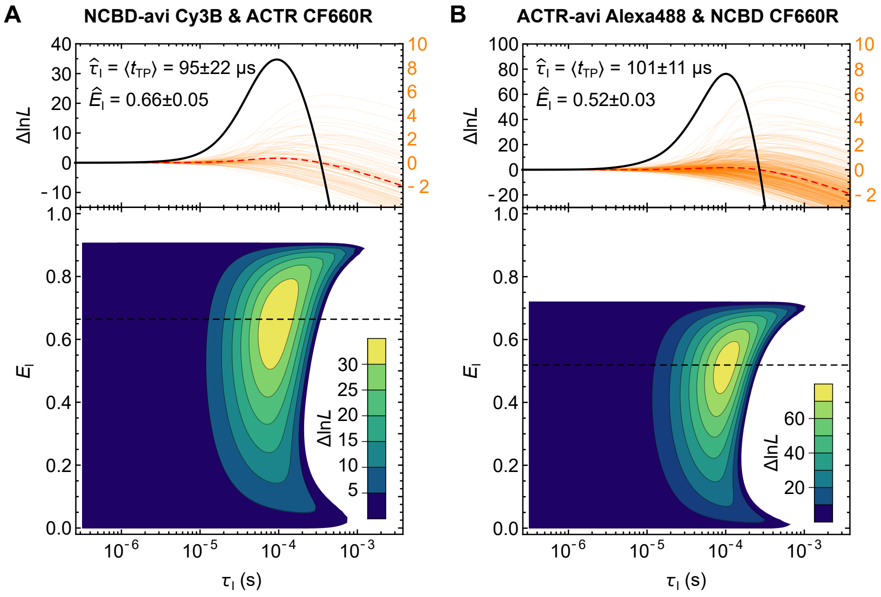

Supplementary Figure 3: Robustness of transition path time measurements to immobilization and dye labeling. (**A**) $\Delta \ln L$ plots for measurements with immobilized Cy3B-labeled NCBD-avi and CF660R-labeled ACTR free in solution. $\langle t_{\mathrm{TP}} \rangle$ and $\widehat{E}_{\mathrm{I}}$ agree with the ones measured with immobilized ACTR within the uncertainty (data from 93 transitions). (**B**) $\Delta \ln L$ plots for measurements with Alexa488-labeled ACTR-avi and CF660R-labeled NCBD. $\langle t_{\mathrm{TP}} \rangle$ is similar to the one for ACTR-avi labeled with Cy3B; $\widehat{E}_{\mathrm{I}}$ is lower, as expected from the smaller Förster radius of the pair Alexa488/CF660R compared to Cy3B/CF660R (data from 440 transitions). Standard errors were obtained from 1000 bootstrapping trials.

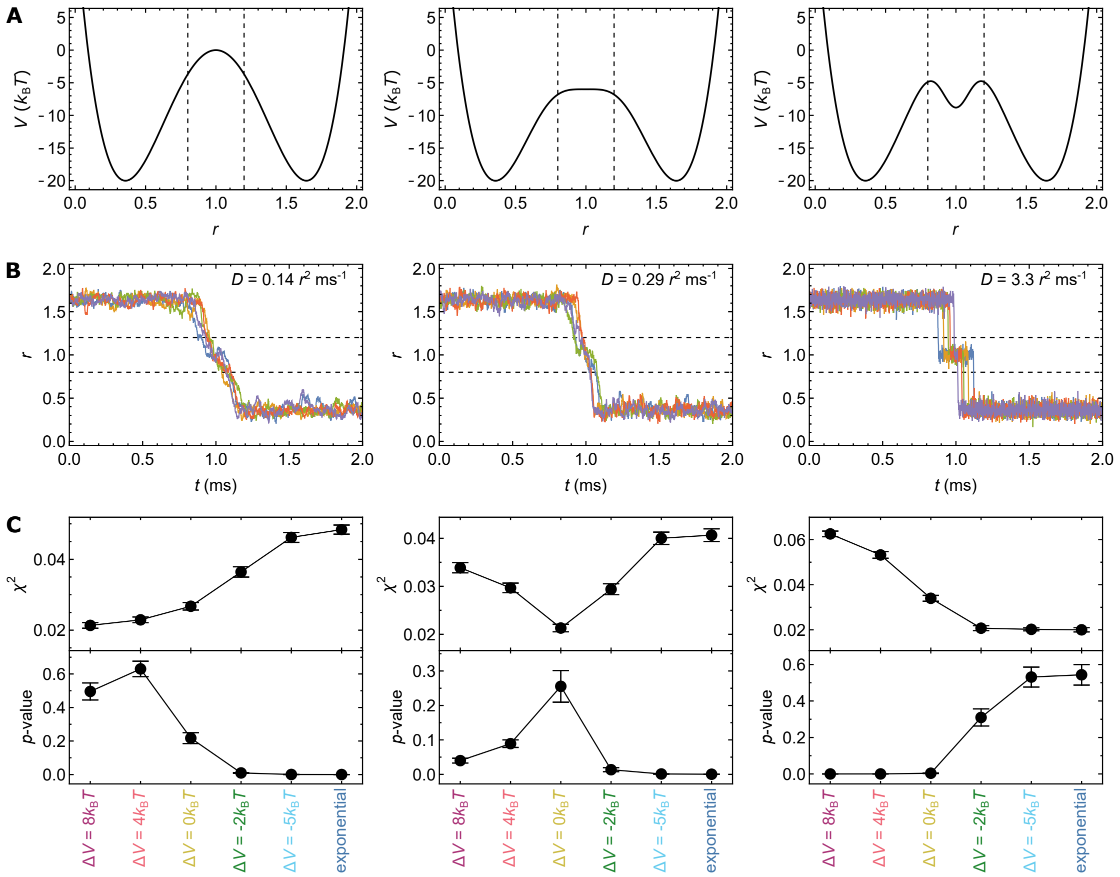

Supplementary Figure 4: Brownian dynamics simulations used to test the method for distinguishing the shapes of the underlying potentials. (**A**) The three different potentials used in the simulations. The dashed lines indicate the boundaries of the transition region. (**B**) Examples of simulated transitions (centered at $t = 1\,\mathrm{ms}$). (**C**) Results of the analysis, showing the χ^2-distances and the p-values from the k-sample Anderson-Darling test for the t_{TP}-distributions shown in Fig.3C (error bars show standard errors from 27 simulations). See Methods for details.

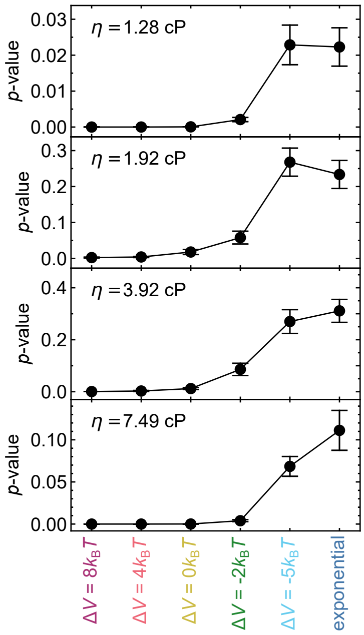

Supplementary Figure 5: Comparing simulated and experimental transition path time distributions with the k-sample Anderson-Darling test, which tests whether measured and simulated samples originate from the same underlying transition path time distribution without specifying the functional form of the distribution. Plotted are the *p*-values for different distributions assumed in the simulations (Fig. 3) for the different solution viscosities (error bars show standard errors from 27 simulations). Higher *p*-values indicate better agreement between observed and simulated distributions and mirror the χ^2-distances shown in Fig. 3.

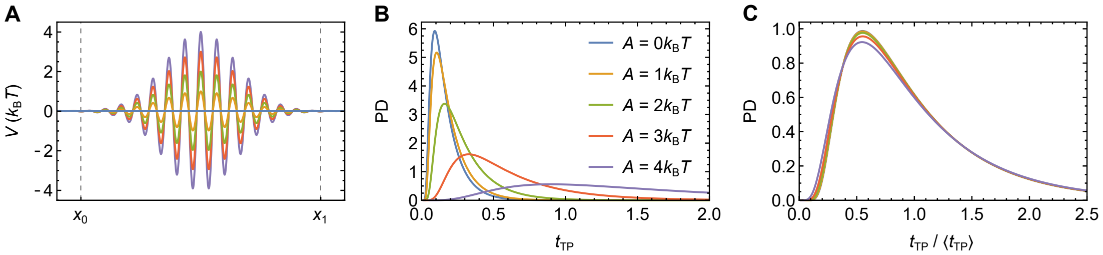

Supplementary Figure 6: Influence of energetic roughness on transition path time (t_{TP}) distributions. (**A**) A flat potential with roughness of different amplitudes was modeled by adding a sinusoidal potential of the form $V(x) = A \cdot \exp(-x^2/0.0225) \cdot \cos(30\pi x)$ to the middle of the transition region (see legend in (**B**) for amplitudes A; transition boundaries are $x_1 = -x_0 = 0.5$). (**B**) t_{TP} distributions resulting from the different amplitudes of roughness in (**A**), calculated as described in "Theoretical transition path time distributions". The timescale is given in units of $(x_1 - x_0)^2/D$, where D is the diffusion coefficient. (**C**) Normalizing the t_{TP} distributions from (**B**) shows that their shapes hardly change with increasing roughness, in contrast to the changes with increasing stability of the intermediate (Fig. 3C).

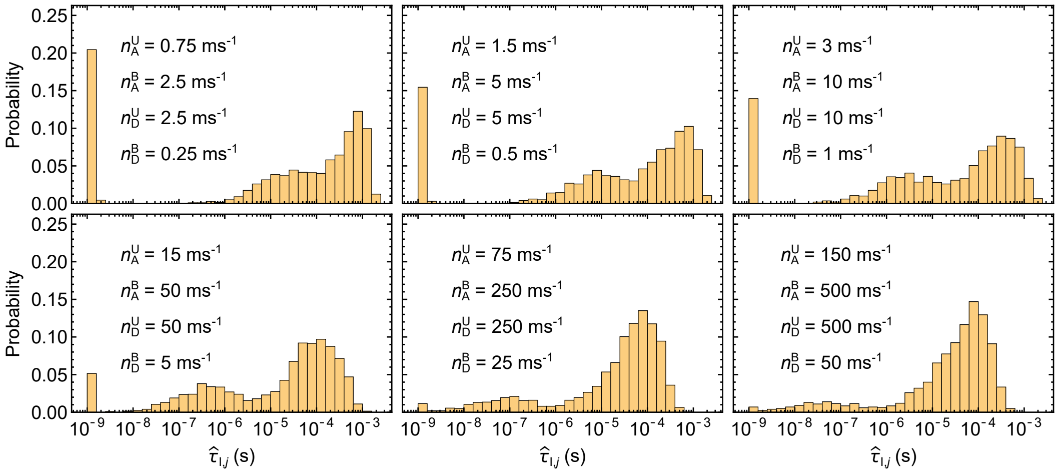

Supplementary Figure 7: Effect of photon count rates on $\widehat{\tau}_{\mathbf{I},j}$ histograms. Photon time traces of binding transitions were simulated as described in "Analysis of transition path time distributions". For each dataset, 2000 transitions were simulated, each with a length of $10\,\mathrm{ms}$ and a duration of the intermediate step drawn randomly from an exponential distribution with a mean of $80\,\mu\mathrm{s}$. The photon count rates used for each dataset (acceptor (A) and donor (D) in bound (B) and unbound (U)) are shown in each plot. The results show a decrease in the peaks at short times with increasing photon rates, suggesting that they are mostly due to transitions that are too fast to be accurately determined at the corresponding photon count rates.

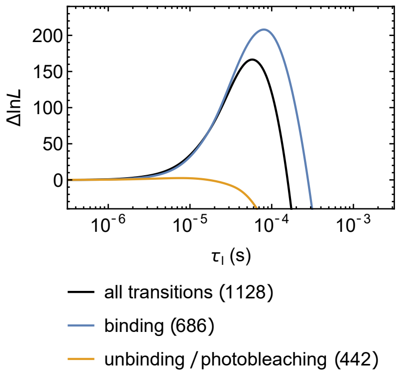

Supplementary Figure 8: Comparison of $\Delta \ln L$ curves of all experimentally observed transitions, and binding and unbinding/photobleaching transitions. Likelihoods were calculated with $E_\mathrm{I} = 0.72$. While the binding transitions show a highly significant peak, the unbinding/photobleaching transitions show no peak, because acceptor photobleaching events (which cannot be distinguished from dissociation events in the experimental data based on the change in transfer efficiency) have a much shorter transition path time. The number of transitions included in each curve is shown in parentheses. The $\Delta \ln L$ curves shown are from the dataset at 108mM ionic strength and at 1.28cP.

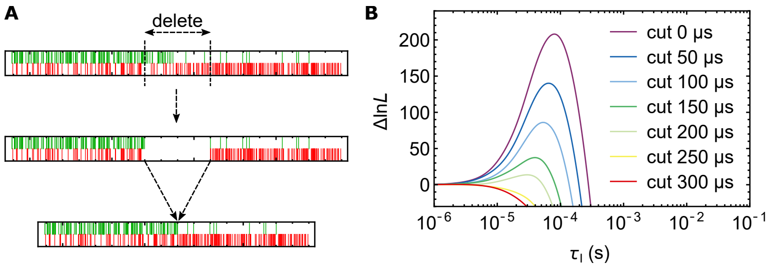

Supplementary Figure 9: Deleting transitions from time traces to mimic instantaneous transitions. (**A**) Photons in the transition region are deleted and the resulting traces before and after the boundaries are joined back together (donor photons are shown in green and acceptor photons in red). (**B**) The resulting $\Delta \ln L$ curves with segments of different lengths deleted show the disappearance of the maximum with increasing segment length, demonstrating that the maximum is caused by the finite duration of the transition paths. The $\Delta \ln L$ curves shown are from the dataset at 108mM ionic strength and at 1.28cP.

Supplementary Tables

Supplementary Table 1: Parameters found from long photon time traces at different ionic strengths

Ionic strength (mM)	Number of time traces	Total time (s)	c_{NCBD} (nM)	$\overline{k}_{\mathrm{on}}$ (s^{-1})	k_{on} ($s^{-1}\,\mu M^{-1}$)	k_{off} (s^{-1})	$k_{+\mathrm{b}}$ (s^{-1})	$k_{-\mathrm{b}}$ (s^{-1})	$\langle E_{\mathrm{B}} \rangle$
51	97	1875	18 ± 1	1.84 ± 0.08	103 ± 8	16.1 ± 0.6	6.2 ± 0.8	260 ± 20	0.882 ± 0.002
108	37	818	30.0 ± 0.5	2.0 ± 0.1	65 ± 4	21.0 ± 0.8	11 ± 3	390 ± 50	0.895 ± 0.004
159	54	1108	39 ± 4	1.70 ± 0.05	44 ± 4	19.5 ± 0.8	4 ± 1	280 ± 30	0.900 ± 0.003
211	51	1072	42.7 ± 0.5	1.45 ± 0.08	34 ± 2	23.1 ± 0.9	3 ± 1	320 ± 60	0.883 ± 0.003
287	64	1268	59 ± 5	2.0 ± 0.4	33 ± 7	24 ± 2	5 ± 2	310 ± 10	0.896 ± 0.002
414	59	1159	65 ± 2	1.3 ± 0.3	20 ± 4	29 ± 1	5 ± 2	330 ± 30	0.888 ± 0.005
914	105	2207	66 ± 1	1.16 ± 0.03	17.6 ± 0.6	20.0 ± 0.6	2.9 ± 0.6	200 ± 30	0.889 ± 0.002

Standard errors are obtained from two independent measurements (c_{NCBD}), from 100 bootstrapping trials ($\overline{k}_{\mathrm{on}}$, k_{off}, $k_{+\mathrm{b}}$, $k_{-\mathrm{b}}$), or from every set of time traces ($\langle E_{\mathrm{B}} \rangle$).

Supplementary Table 2: Transfer efficiencies of the bound state determined from long photon time traces for different variants and at different viscosities

Variants	Viscosity (cP)	Number of time traces	$\langle E_{\mathrm{B}} \rangle$
ACTR-avi Cy3B, NCBD CF660R	1.28	181	0.899 ± 0.002
	1.92	155	0.899 ± 0.002
	3.92	100	0.887 ± 0.003
	7.49	39	0.873 ± 0.004
NCBD-avi Cy3B, ACTR CF660R	1.28	42	0.907 ± 0.003
ACTR-avi Alexa488, NCBD CF660R	1.28	136	0.719 ± 0.004

Averages and standard errors from every set of time traces.

Supplementary Table 3: Parameters obtained from high-intensity photon time traces

Ionic strength (mM)	$\widehat{\tau}_{\mathrm{I}} = \langle t_{\mathrm{TP}} \rangle$ (μs)	$\widehat{E}_{\mathrm{I}}$	Number of transitions analyzed	Average total photon rate (ms^{-1})	Sizes of analyzed windows (ms) low-mean-high
51	110 ± 20	0.71 ± 0.03	191	155 ± 4	2-11-41
108	80 ± 8	0.71 ± 0.02	686	199 ± 4	1-9-41
159	80 ± 20	0.72 ± 0.05	79	270 ± 8	1-5-11
211	100 ± 10	0.73 ± 0.02	257	184 ± 4	2-8-28
287	90 ± 10	0.75 ± 0.02	379	146 ± 2	2-9-41
414	90 ± 10	0.70 ± 0.02	205	170 ± 5	2-10-41
914	100 ± 20	0.71 ± 0.03	184	142 ± 4	1-9-41
Viscosity (cP)					
1.28	80 ± 8	0.71 ± 0.02	686	199 ± 4	1-9-41
1.92	140 ± 20	0.71 ± 0.02	331	104 ± 1	3-14-41
3.92	440 ± 40	0.73 ± 0.01	285	74 ± 2	5-21-91
7.49	680 ± 50	0.69 ± 0.02	378	44 ± 1	7-46-165
Variants					
NCBD-avi Cy3B, ACTR CF660R	95 ± 22	0.66 ± 0.05	93	162 ± 5	2-11-41
ACTR-avi Alexa488, NCBD CF660R	101 ± 11	0.52 ± 0.03	440	112 ± 2	3-13-82

Standard errors are obtained from 1000 bootstrapping trials ($\widehat{\tau}_{\mathrm{I}}$ and $\widehat{E}_{\mathrm{I}}$) and from every set of time traces (average total photon rates and sizes of analyzed windows).

Supplementary Table 4: Diffusion coefficients resulting in the best agreement between calculated and measured transition path time distributions for the different shapes of the potential (Fig.3)

Viscosity (cP)	ΔV ($k_\mathrm{B}T$)	D/D_max
1.28	8	0.018
	4	0.028
	0	0.059
	-2	0.14
	-5	1.
1.92	8	0.010
	4	0.015
	0	0.034
	-2	0.075
	-5	0.53
3.92	8	0.010
	4	0.0033
	0	0.0050
	-2	0.010
	-5	0.024
7.49	8	0.0021
	4	0.0034
	0	0.0072
	-2	0.016
	-5	0.012

Diffusion coefficients D are given relative to the highest diffusion coefficient, D_max. For each barrier shape, D has to be adjusted so that the simulated $\widehat{\tau}_\mathrm{I}$ matches the measured $\widehat{\tau}_\mathrm{I}$. For the parabolic barriers, e.g., diffusion needs to be slower than in the presence of an intermediate for best agreement.
